# A role for NRAGE in NF-κB activation through the non-canonical BMP pathway

**DOI:** 10.1186/1741-7007-8-7

**Published:** 2010-01-25

**Authors:** Nicholas Matluk, Jennifer A Rochira, Aldona Karaczyn, Tamara Adams, Joseph M Verdi

**Affiliations:** 1 Maine Medical Center Research Institute, Center for Molecular Medicine, 81 Research Drive, Scarborough, Maine 04074, USA; 2 Graduate School of Biomedical Sciences, The University of Maine at Orono, 267A Engineering and Science Research Building, Orono, Maine, 04469 USA; 3 Functional Genomics IGERT Program, 263 Engineering and Research Science Building, The University of Maine at Orono, Orono, Maine, 04469 USA

## Abstract

**Background:**

Previous studies have linked neurotrophin receptor-interacting MAGE protein to the bone morphogenic protein signaling pathway and its effect on p38 mediated apoptosis of neural progenitor cells via the XIAP-Tak1-Tab1 complex. Its effect on NF-κB has yet to be explored.

**Results:**

Herein we report that NRAGE, via the same XIAP-Tak1-Tab1 complex, is required for the phosphorylation of IKK -α/β and subsequent transcriptional activation of the p65 subunit of NF-κB. Ablation of endogenous NRAGE by siRNA inhibited NF-κB pathway activation, while ablation of Tak1 and Tab1 by morpholino inhibited overexpression of NRAGE from activating NF-κB. Finally, cytokine profiling of an NRAGE over-expressing stable line revealed the expression of macrophage migration inhibitory factor.

**Conclusion:**

Modulation of NRAGE expression revealed novel roles in regulating NF-κB activity in the non-canonical bone morphogenic protein signaling pathway. The expression of macrophage migration inhibitory factor by bone morphogenic protein -4 reveals novel crosstalk between an immune cytokine and a developmental pathway.

## Background

The bone morphogenic proteins (BMPs) are a subset of the transforming growth factor β (TGF-β) superfamily and function through the dimerization of the BMPR-1a and BMPR-2a serine threonine kinase receptors. The canonical BMP pathway regulates gene expression via SMAD1, 4, 5, and 8 [1] while the non-canonical BMP pathway regulates NF-κB via the XIAP-Tak1-Tab1 complex [2,3]. Both pathways help direct proper proliferation and differentiation, embryogenesis and adulthood.

The NF-κB pathway is activated at sites of injury and controls the expression of inflammatory and immune regulating cytokines and chemokines as well as regulating apoptosis and cell cycle. The NF-κB pathway consists of several transcription factors which are bound as homodimers or heterodimers; p65 (RelA), p50 (NF-kB1), p52 (NF-kB2), RelB, and c-Rel [4,5]. These transcription factors reside in an inactive state in the cytoplasm bound to the IκB family of proteins. Activation with various stimuli including but not limited to LPS, flagellin, Lipid A, ssRNA, dsRNA and cytokines causes them to be translocated to the nucleus [4,5]. The canonical NF-κB pathway requires IKKαβγ phosphorylation and subsequent degradation of IkBα. Previous research has linked the canonical NF-κB pathway to the non-canonical BMP pathway via the formation of the XIAP-Tab1-Tak1 complex [2,3,6-8].

Adapter proteins play a pivotal role in the regulation and function of signal transduction pathways. Activation of NF-κB pathways through the stimulation of toll-like receptors (TLRs) or interleukin pathways require MyD88, IRAK proteins, and TRAF proteins to propagate the signal from the extracellular matrix to the Tak1-Tab1 complex which is responsible for the phosphorylation of IKK-α/β. The non-canonical BMP pathway also uses the Tak1-Tab1 complex to drive phosphorylation of IKK-α/β, but uses the ring finger protein XIAP instead of TRAF6 [2,3,6-8]. The similarity of these two pathways suggested that an additional adapter protein could be present linking XIAP-Tak1-Tab1 to the BMPR1a receptor.

A member of the melanoma antigen family, the neurotrophin receptor-interacting MAGE protein (NRAGE) is ubiquitously expressed in tissue and contains a unique WQXPXX hexapeptide repeat domain, suggesting that NRAGE has a unique function which differs from the other MAGE family of proteins. Previous studies linked NRAGE to XIAP using a yeast two hybrid screen [9] while we have previously identified NRAGE as a critical component in the activation of p38 and subsequent downstream proapoptotic signals via the non-canonical BMP pathway [10,11]. Using a similar approach to Kendall et al. in which the expression of NRAGE was modulated through a series of loss of function and gain of function experiments, we have found that NRAGE is also a required component in driving NF-κB activation through the BMPR1a-XIAP-Tak1-Tab1 complex in 293HEK cells.

## Results

### NRAGE is required for NF-κB activation in the non-canonical BMP-4

**Figure 1:**
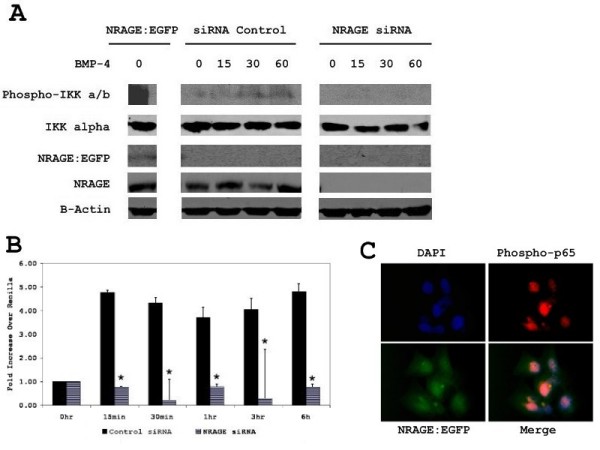
**Activation of the NF-κB pathway requires NRAGE in the BMP-4 pathway**. **A**: A total of 293 cells were transfected with NRAGE:EGFP, NRAGE siRNA or a control vector, stimulated with 10 ng/ml BMP-4 for 15, 30 and 60 minutes, and western blotted for NRAGE, phospho-IKK α/β and total IKK α. **B**: A total of 293 cells were transfected with NRAGE siRNA or a control siRNA, stimulated with 10 ng/nl BMP-4, and activation assessed by luciferase assay. Luciferase assays were performed in triplicate and presented as fold increase over renilla. **C**: Immunofluorescence of phospho-p65 in the NRAGE:EGFP stable line. Magnification at 40×. * *P *< 0.05; siRNA control by the two-tailed unpaired Student's *t *test.

Previous reports linked NRAGE to the XIAP-Tab1-Tak1 complex and phosphorylation of p38 [10,11]. We wanted to determine if NRAGE was also required for the activation of NF-κB through the non-canonical BMP pathway. Overexpression of full length NRAGE in 293HEK cells resulted in the constitutive phosphorylation of IKK-α and IKK-β (Figure 1A) while disruption of NRAGE expression through siRNA resulted in the inhibition of IKK-α/β phosphorylation (Figure 1A). When compared to cells transfected with control siRNA, the NRAGE siRNA transfected cells showed an ablation in transcriptional activation of the p65 subunit of NF-κB after treatment with BMP-4 (Figure 1B). Stimulation with BMP-4 or transfection with NRAGE resulted in translocation of p65 to the nucleus as verified by immunofluorescence (Figure 1C). Transfection of 293HEK cells with NRAGE siRNA prior to stimulation with BMP-4 abrogated this effect as p65 was still found sequestered in the cytoplasm (Figure 1C).

**Figure 2:**
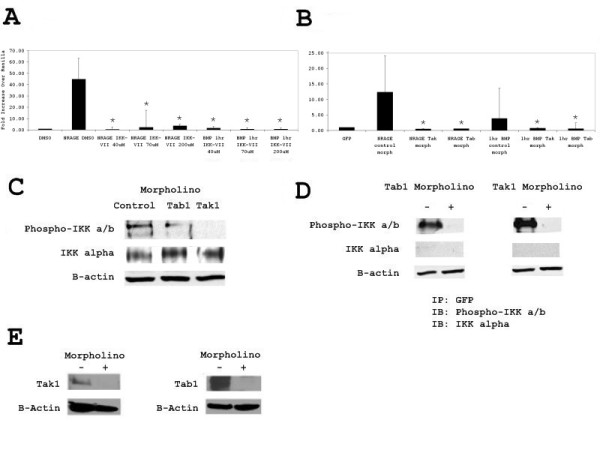
**Activation of NF-κB through NRAGE is specific to the non-canonical BMP pathway**. **A**: 293 cells were transfected with full length NRAGE:EGFP or stimulated with BMP-4 and incubated with or without IKK VII inhibitor, and NF-κB activation assessed by luciferase assay. **B**: 293 cells were transfected with full length NRAGE:EGFP or stimulated with BMP-4 and incubated with or without Tak1 or Tab1 morpholinos, and NF-κB activation assessed by luciferase assay. **C**: Endogenous phosphorylation of IKK-α/β in 293 cells transfected with Tak1 or Tab1 morpholinos prior to 10 ng/ml BMP-4 stimulation for one hour. **D**: 293 cells were transfected with full length NRAGE:EGFP, incubated with or without Tak1 or Tab1 morpholinos immunoprecipitated with rabbit anti-GFP and western blotted for IKK α/β-phosphorylation and total IKK α. **E**: Western blot of Tak1 and Tab1 proteins in 293 cells with and without morpholinos. Luciferase assays were performed in triplicate and presented as fold increase over renilla. * *P *< 0.05; vs NRAGE construct or BMP-4 treatment by the two-tailed unpaired Student's *t*-test.

To ensure that the activity of NRAGE on the NF-κB pathway was specific to the non-canonical BMP pathway, the inhibitor IKK-VII was used to eliminate the phosphorylation of IKK-α and IKK-β and IKK-γ and subsequent ubiquitination of IκBα. IKK-VII is a concentration dependent specific ATP competitive inhibitor of IKK proteins. Inhibition of the IKK proteins resulted in a decrease in p65 transcriptional activity in the NRAGE transfected cells (Figure 2A). Stimulation of GFP transfected with BMP-4 served as an internal control (Figure 2A).

Previous work has shown that a Tak1 or Tab1 morpholino is sufficient for ablation of p38 activity in cells overexpressing NRAGE [10,11]. Similar to this, we found that NF-κB transcriptional activation (Figure 2B) and IKK-α/β phosphorylation (Figure 2C) were ablated in 293HEK cells overexpressing NRAGE when treated with Tak1 or Tab1 morpholinos for 48 hours. Figure 2C depicts endogenous phospho-IKK-α and phospho-IKK-β after stimulation with BMP-4 and after incubation with Tak1 or Tab1 morpholinos for 48 hours.

Immunoprecipitation studies using rabbit anti GFP to purify protein complexes involving NRAGE revealed that phospho-IKK-α and phospho-IKK-β are bound upon overexpression of NRAGE:GFP fusion protein (Figure 2D). Incubation of NRAGE:GFP transfected cells with either Tak1 or Tab1 morpholinos for 72 hours prevented the formation of the phospho-IKK-α/β and NRAGE complex (Figure 2D). The efficiency of Tak1 and Tab1 morpholinos are shown in Figure 2E.

### Overexpression of NRAGE results in the expression of macrophage migration inhibitory factor

**Figure 3:**
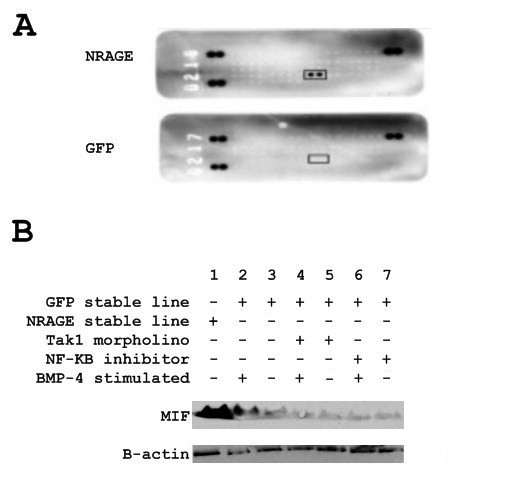
**BMP-4 induces the expression of MIF**. **A**: Dot blot array for the secretion of cytokines and chemokines induced by NRAGE overexpression in 293HEK cells. Squared area indicates duplicate MIF blotting. **B**: Qualification of macrophage migrating inhibitory factor expression by western blot in 293HEK cells. MIF expression is induced after 24 hours stimulation with 10 ng/ml BMP-4 or NRAGE overexpression, and is inhibited by Tak morpholino and NF-κB inhibitor.

Although a primary result of non-canonical BMP signaling is apoptosis, we surmise that there is an anti-apoptotic component to this pathway since there is a niche of neural stem cells in the adult brain despite BMP induced apoptosis of neural stem cells during development [12-14]. Analysis of 293HEK cells stably transfected with either GFP or the adapter protein NRAGE:GFP using the Human Cytokine Protein Array (R&D Systems, Minneapolis, Minnesota, United States, Cat: ARY005), revealed that macrophage migration inhibitory factor (MIF) was highly expressed (square box Figure 3A). Analysis of the 293HEK EGFP stable line stimulated with and without BMP-4 was used to determine endogenous expression of MIF (Figure 3B, Lane 2 and 3). MIF expression of the 293HEK NRAGE:EGFP stable line is shown in lane 1 of Figure 3B. Ablation of MIF expression occurred when Tak1 morpholino was added, prior to BMP stimulation (Figure 3B, Lane 4 and 5) and when NF-κB inhibitor was added prior to BMP-4 stimulation (Figure 3B, Lane 6 and 7).

### Overexpression of NRAGE in the mouse kidney

**Figure 4:**
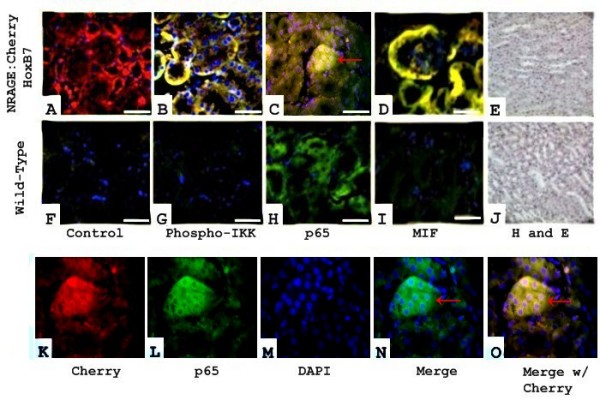
***In vivo *overexpression of NRAGE in the mouse kidney**. **A**: Control staining of secondary antibodies in the NRAGE:Cherry transgenic mouse. **B**: Merged picture of NRAGE:Cherry and IKKα/β-phosphorylation. **C**: Merged picture of NRAGE:Cherry and NF-κB activation. Note the translocation of NF-κB to the nucleus denoted by teal colored nuclei. **D**: Merged picture of NRAGE:Cherry and MIF. **E**: Hematoxylin and Eosin staining of NRAGE:Cherry transgenic kidney. **F**: Control staining for secondary antibodies in the wild type mouse. **G**: Staining of IKKα/β-phosphorylation in the wild type mouse. **H**: Staining of NF-κB activation in the wild type mouse. **I**: Merged picture of NRAGE:cherry and MIF in the wild type mouse. **J**: Hematoxylin and Eosin staining of Wild-Type kidney. **K-O**: 63× magnification of C, red arrows point to nuclear localization of p65 as depicted by teal coloring.

Next, we used mice overexpressing a NRAGE:Cherry fusion protein in the kidney under the control of the HoxB7 promoter, to determine if NF-κB would be constitutively active *in vivo*. Histological analysis revealed that there was constitutive phosphorylation of IKK-α/β (Figure 4B) and translocation p65 to the nucleus (Figure 4C, 4K-4O) in the NRAGE:Cherry transgenic mouse. There was no detected phosphorylation of either IKK-α/β in the wild type kidneys (Figure 4G) or nuclear translocation of the p65 subunit of NF-κB (Figure 4H). Histological analysis of the kidney from the overexpressing NRAGE:Cherry transgenic mouse revealed that there was a dramatic increase in MIF expression detected by Alexa Flour 488 (Figure 4D), as compared to the wild-type kidney (Figure 4I). *In vivo *overexpression of NRAGE does not result in the influx of inflammatory cells (Figure 4E and 4J) as seen by hematoxylin and eosin staining.

### NRAGE induces NF-κB activation and MIF expression in P19 cells

**Figure 5:**
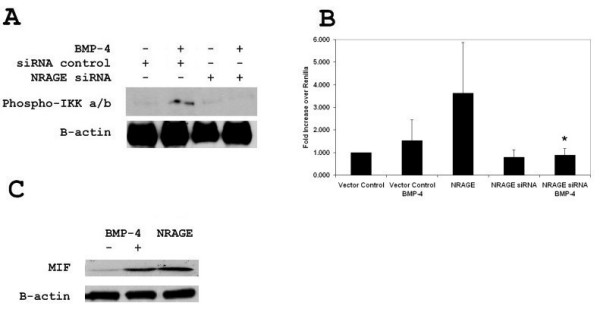
**NRAGE is required for NF-KB activation in P19 cells**. **A**: P19 cells were transfected with NRAGE siRNA or a control siRNA stimulated with 10 ng/ml BMP-4 for 24 hours, and western blotted for IKK α/β-phosphorylation and total IKK α. **B**: P19 cells were transfected with NRAGE, NRAGE siRNA or a control siRNA, stimulated with 10 ng/nl BMP-4 for 24 hours, and NF-KB transcriptional activity assessed by luciferase assay. Luciferase assays were performed in triplicate and presented as fold increase over renilla. **C**: P19 cells were transfected with NRAGE or stimulated with 10 ng/nl BMP-4 for 24 hours and western blotted for MIF expression. * *P *< 0.05; vs NRAGE construct or BMP-4 treatment by the two-tailed unpaired Student's *t*- test.

Experiments were conducted in P19 cells to demonstrate that the results obtained using 293 cells are physiologically relevant. Overexpression of full length NRAGE in P19 cells resulted in the constitutive phosphorylation of IKK-α/β while disruption of NRAGE expression through siRNA resulted in the inhibition of IKK-α/β phosphorylation (Figure 5A). Transfection of P19 cells with NRAGE, renilla and a NF-κB-luciferase reporter vector showed transcriptional activation of the p65 subunit of NF-κB (Figure 5B). Ablation of NRAGE expression with the NRAGE siRNA disrupted NF-κB transcriptional activity (*P*-value of 0.027). IKK-α/β phosphorylation and luciferase expression were ablated when NRAGE siRNA was transfected prior to BMP-4 stimulation. As was seen in Figure 3B, BMP-4 stimulation or NRAGE transfection resulted in an increase in MIF expression (Figure 5C).

## Discussion

Stimulation of cells with BMP-4 activates the canonical and non-canonical BMP pathways. Signal transduction through the canonical pathway utilizes SMAD proteins, while the non-canonical pathway utilizes XIAP-Tab1-Tak1, ultimately using the kinase activity of Tak1 to activate the p38, JNK, and NF-κB pathway. Signal transduction of NF-κB through the non-canonical BMP pathway is similar to the toll-like receptor pathways and interleukin-1 pathway which require the formation of TRAF6-Tab1-Tak1. Both TRAF6 and XIAP are ring finger proteins which dimerize and consist of three baculovirus IAP repeat domains required for proper orientation of the Tab1-Tak1 complex. The difference is that the TLR and IL-1 pathways also utilize several adapter molecules linking the TRAF6-Tab1-Tak1 complex to their membrane bound receptors such as, Tram, Tirap, MyD88, and IRAK [4,5]. Because NRAGE has already been identified as an adapter protein for p38 signaling through the non-canonical BMP pathway, we thought it feasible that NRAGE also plays a role in NF-κB signaling.

Experiments in which cells were transfected with a full length NRAGE expression vector illustrated that IKKαβ became phosphorylated (Figure 1A), induced luciferase expression (Figure 2A and 2B) and p65 translocated to the nucleus (Figure 1C). Experiments in which the expression of NRAGE, Tab1, or Tak1 were reduced, either by siRNA or morpholino showed that all three proteins were required for NF-κB pathway activation (Figures 1A, B and 2B, C, D). It was also found that despite overexpression of NRAGE, silencing of Tab1 or Tak1 inhibited NF-κB activation (Figure 2A), showing that a reduction in downstream kinase protein concentration is able to overcome an increase in upstream protein concentration. Immunoprecipitation of the activated non-canonical BMP complex revealed that NRAGE and phosphorylated IKKα/β but not IKKα are indirectly bound through Tab1 and Tak1 (Figure 2D).

Taken together, these observations support a mandatory role for NRAGE in NF-κB signalling through the non-canonical BMP pathway.

Tak1 is the functional kinase in the non-canonical BMP pathway as well as in interleukin, toll-like receptor and TGF-β signaling pathways; responsible for the phosphorylation and activation of p38, NF-κB, and JNK, and is found complexed together in the cytoplasm with Tab1. Figure 2C shows that siRNA knockdown of Tak1 but not Tab1 results in complete inhibition of IKKα/β phosphorylation. This observation is similar to experiments by Shim et al., which show that NF-κB activation in embryonic fibroblasts from Tak1 -/- mice but not Tab1 -/- and Tab2 -/- mice is perturbed. It is known that Tab2 and Tab3 also associate with Tak1 and Tab1 to regulate the classical NF-κB pathway. Redundancy in function or protein:protein interactions may compensate for the loss or inactivation of one or more of the Tab proteins.

It is interesting to note that NRAGE influences both apoptosis and NF-κB signaling through two distinct mechanisms in two different pathways. In the non-canonical BMP pathway, NRAGE binds XIAP, sequestering it to the cell membrane resulting in the activation of MAPK and NF-κB. While in response to DNA damage, NRAGE prevents the expression of XIAP via NF-κB by sequestering Che-1 in the cytoplasm where it is targeted for ubiquitination and degradation [15,16]. Does this constitute an intrinsic link between NRAGE/XIAP/NF-κB and apoptosis? And if so will NRAGE be found to regulate other NF-κB pathways such as the toll-like receptors where XIAP is upregulated upon TLR-4 stimulation [17]?

Protein array analysis of secreted cytokines from the NRAGE:EGFP stable line revealed that macrophage migration inhibitory factor (MIF) was highly expressed (Figure 3A) and western blot of protein lysates confirmed MIF expression in the cytoplasm. Stimulation of the EGFP stable line with BMP-4 for 24 hours revealed that MIF expression is specific to the BMP pathway, while ablation of Tak1 detail that MIF is being induced through the non-canonical BMP pathway (Figure 3B). The MIF promoter contains two NF-κB binding sites, located at positions -2538/-2528 bp and -1389/-1380 bp [18], inhibition of IKK prior to BMP-4 stimulation prevented the expression of MIF (Figure 3B).

Classical research into MIF has focused on its regulatory role of immune system [19]. Recent findings though indicate that MIF can control the cell cycle through interactions with JAB1 and p27^KIP1 ^[20], control proliferation [19], and inhibit apoptosis through the stabilization of p53 and Mdm2 [21,22]. Constitutive activation of the BMP pathway [23-26] and the expression of MIF [27-30] have been linked to a variety of cancers.

The BMP pathway is required for proper kidney development and function and injury repair [31-35]. MIF is constitutively expressed in the kidney [36] and is upregulated in chronic kidney disease, glomerulonephritis, and oxidative stress [37-40]. Nikopoulos et al. used transgenic mice in which a NRAGE:Cherry fusion protein is under control of the HoxB7 promoter and results in constitutive phosphorylation of p38^MAPK ^in the kidney through the non-canonical BMP signaling pathway [11]. Our immunohistological analysis of these transgenic kidneys revealed that there was also constitutive activation of the NF-κB pathway (Figure 4B-C) and an increase in MIF expression (Figure 4D). It would be interesting to determine if BMP induces MIF expression in response to both renal development and injury, counteracting BMP induced renal apoptosis.

BMP expression is present in neural stem cell migratory pathways and the neural crest during embryogenesis, controlling neural crest differentiation and apoptosis in the hindbrain. On mouse embryonic Day 13, when corticogenesis begins, BMP-2 and 4 expression are dramatically increased, affecting neural stem cells in the ventricular zone, inhibiting their proliferation through apoptosis [41-43]. Eventually, two neural stem cell niches are established having escaped BMP induced apoptosis; one in the subventricular zone (SVZ) and one in the subgranular zone (SGZ). We used the mouse embryonic carcinoma P19 cell line a model for BMP induced apoptosis in neural stem cells [44], to illustrate that the BMP NRAGE NF-κB link is not just restricted to 293HEK cells (Figure 5). With the novel finding that MIF expression can be linked to BMP signaling and that MIF is expressed during embryogenesis [45-48], it is imperative that the correlation between the anti-apoptotic cytokine MIF and BMP driven apoptosis be elucidated.

## Conclusion

The finding that NRAGE regulates NF-κB signaling in the non-canonical BMP pathway adds an extra level of control to an already highly regulated developmental pathway. The observation that stimulation of the non-canonical BMP pathway results in MIF expression, represents novel crosstalk between a classical immune cytokine and a developmental pathway which requires future consideration.

## Methods

### Cell culture and transfection

293HEK cells were cultured in DMEM:F12 media supplemented with 10% FBS, gentamycin at 37°C 5% CO_2_. Cells were trypsinized and passaged at 75% confluency. Genejuice (EMDBiosciences, Darmstadt, Germany) was used per manufacturers' instructions for the transfection of plasmids into 293HEK cells and Endoporter (Gene-Tools, Philomath, OR, USA) was used per manufacturer's instructions for the transfection of morpholinos. Cells transfected with siRNA, shRNA or morpholinos were cultured for at least 48 hours prior to analysis. Cells were exposed to IKK-VII (EMDBiosciences, Cat. 410486) at varying concentrations throughout the entire procedure to inhibit NF-κB activation. In all experiments, cells were serum starved for approximately four hours prior to the addition of 10 ng/ml of BMP-4 (R&D Systems, Minneapolis, MN, USA).

### Plasmids and morpholinos

Full length NRAGE was cloned into the mammalian expression vector pEGFP-N3 (Clonetech, Mountain View, CA, USA, Cat. PT3052-5). The pEGFP-N3 vector codes of the neomycin resistance gene and was used to aid in the creation of a NRAGE:EGFP stable line. NRAGE siRNA Sense GGCUUGGAAUGACACUACUtt and Anti Sense AGUAGUGUCAUUCCAAGCCtt (Ambion, Austin, TX, USA) were cloned into psuppressor.retro (Imgenex, San Diego, CA, USA). Control siRNA #1 was supplied by Ambion (Cat. 4611). The following morpholinos were used: NRAGE morpholino GGTTTCTGAGCCATAGCTCTCGTC (Gene-Tools), Tak1 morpholino AGCGCCCTTCAGCCCGGAGCCC (Gene-Tools), Tab1 morpholino CAGGCTCCTCCTCTGCGCCGCCATC (Gene-Tools), Control morpholino CCTCTTACCTCAGTTACAATTTATA (Gene-Tools).

### Western blotting and immunoprecipitation

Cells were serum starved for approximately four hours prior to the addition of 10 ng/ml of BMP-4 (R&D Systems) or cells were transfected with respective vectors for 24 hours prior to being lysed with either RIPA buffer or NP-40 buffer supplemented with tyrosine phosphatase inhibitors (Upstate, Billerica, MA, USA, Cat. 20-203), Ser/Thr phosphatase inhibitors (Upstate, Cat.20-204) and Protease Inhibitor Cocktail Set III (Calbiochem, Darmstadt, Germany, Cat. 539134). Lysates were then centrifuged at 10,000 rpm and the supernatant stored at -80°C until use. Protein concentration was obtained via Pierce BCA Kit (Rockford, IL, USA, Cat. 23235) per manufacturer's instructions. Western blotting and immunoprecipitation were performed as previously described [44]. The following antibodies were used for western blotting: rabbit anti-phospho-IKK α/β (Cell Signaling, Danvers, MA, USA, Cat. 2078) (1:1000 dilution), rabbit anti-IKK α (Cell Signaling, Cat. 2682) mouse anti β-actin (Novus, Littleton, CO, USA, Cat. NB 600-501) (1:10000 dilution), mouse anti-Tak1 (Santa Cruz, Santa Cruz, CA, USA, Cat. sc-7967) (1:1000 dilution), goat anti-Tab1 (Santa Cruz, Cat. sc-6052) (1:1000 dilution), goat anti-NRAGE (Santa Cruz, Cat. sc-14400 and sc-14398) (1:500 dilution), rabbit anti-GFP (Santa Cruz, Cat. sc-8334) (1:1000 dilution), and rabbit anti-MIF (Santa Cruz, Cat. sc-20121) (1:1000 dilution). The following secondary antibodies were all diluted to 1:3000; goat anti-rabbit HRP (Bio-Rad, Hercules, CA, USA, Cat. 170-6515), goat anti-mouse HRP (Bio-Rad, Cat. 170-6516), and donkey anti-goat HRP (Santa Cruz, Cat. sc-2020). Analysis of cytokine expression on the EGFP and NRAGE:EGFP stable line using the Human Cytokine Protein Array (R&D Systems, Cat: ARY005) was performed as per manufacturer's instructions.

### Luciferase assay

293HEK cells in a 100 mm dish were first transfected with NF-κB luciferase (Stratagene, LaJolla, CA, USA, Cat. 219078-51) and Renilla via Genejuice (EMDBiosciences). These cells were then trypsinized, counted and plated at a density of 30,000 cells/well in 24 well culture plates, prior to transfection with various plasmids using Genejuice. The Dual Luciferase Assay Kit (Promega, Madison, WI, USA, Cat. E1980) was used for the analysis of NF-κB activation. All data are presented as a fold increase over renilla activity and were performed in triplicate.

### Immunohistochemistry

Six-month-old wild type and NRAGE:Cherry HoxB7 mice were euthanized via CO_2 _asphyxiation, kidneys dissected and fixed with 4% PFA. Kidneys were then embedded into paraffin, 5 μm sections cut, deparaffined with xylene and rehydrated in decreasing amounts of alcohol. Sections were blocked with 5% BSA, 0.1% goat serum, in TBST for one hour at room temperature. Sections were incubated with the following primary antibodies for two hours at room temperature in blocking buffer: mouse anti NFKB p65 (Transduction Laboratories, BdBiosciences, San Jose, CA, USA, Cat. N67620-050) (1:10 dilution), rabbit anti-phospho-IKK α/β (Cell Signaling, Cat. 2078) (1:10 dilution), rabbit anti MIF (Santa Cruz, Cat. sc-20121) (1:50 dilution), rabbit anti-phospho-p65 (Cell Signaling, Cat. 3033) (1:50 dilution). After washing with PBS, the sections were then incubated for 30 minutes at room temperature with goat anti-mouse Alexa fluor 488 (Molecular Probes, Carslbad, CA, USA, Cat. A-11029) (1:1000) or goat anti rabbit Alexa fluor 488 (Molecular Probes, Cat. A-11008) (1:1000) in blocking buffer. Dual primary antibody staining was performed in sequential fashion. The cells were then washed with PBS, stained with DAPI and stored at 4°C. Images were taken on a Zeiss Axiovert 200 fluorescent microscope and pseudo colored with MetaMorph software version 6.1 (Universal Imaging Corporation, Sunnyvale, CA, USA).

### NRAGE:Cherry HoxB7 transgenic mice

Creation and maintenance of transgenic mice is as previously described [11]. Protocols and procedures were approved by MMCRI's IACUC; under the title of Numb/NRAGE in tumor Metastasis, project number 0801.

## Abbreviations

BMP: bone morphogenic protein; GFP: green fluorescent protein; MIF: macrophage migration inhibitory factor; NRAGE: neurotrophin receptor-interacting MAGE protein; TGF-β: transforming growth factor β.

## Competing interests

The authors declare that they have no competing interests.

## Authors' contributions

NM designed, performed or assisted on all experiments. JR constructed the NRAGE plasmid used and AK established the NRAGE stable line. TA assisted with mouse dissections and histology slides. All authors provided a detailed examination and critique of the manuscript prior to submission.

## Authors' information

NM is a PhD candidate in cellular biology with a focus in developmental immunology. JR is a PhD candidate in functional genomics with a focus in neural apoptosis. JV is the primary investigator whose research is focused on neural development.
